# Research on the effect of TMS on insomnia patients: EEG changes and prognostic modeling

**DOI:** 10.3389/fnins.2025.1586509

**Published:** 2025-10-13

**Authors:** Jiaxiu He, Jin-xiang Cheng, Changjun Su, Jun Zhang

**Affiliations:** Department of Neurology, Tangdu Hospital, Xi'an, China

**Keywords:** insomnia, EEG analysis, artificial intelligence, sleep disorder, support vector regression

## Abstract

**Objective:**

Insomnia (ID) is the most common clinical disorder afflicting people of all ages, races, and social classes. This study was to explore changes in the brain’s nervous system of insomnia patients after TMS treatment and construct a prognostic prediction model.

**Method:**

This study involved collecting EEG data of 15 patients before and after treatment, extracting features (approximate entropy, sample entropy, alignment entropy, power spectral density, median, mean, kurtosis, and skewness), and building an SVR model.

**Results:**

Fifteen subjects (8 females, 7 males, mean age 42 years) received 7 days of TMS on the right prefrontal lobe. Five eigenvalues were used to analyze EEG data in 5 frequency bands. Statistically significant indicator eigenvalues (*p* < 0.05). Paired *t*-test showed significant differences in PSQI and ISI total scores before and after TMS treatment, indicating its therapeutic effect. Correlation coefficients between 40 indicators and scale differences were calculated, and significant characteristic values were further analyzed. SVR models for predicting ISI and PSQI scale pre-post differences were constructed. Both had predictive ability.

**Conclusion:**

This work proposes the first SVR model leveraging pre-treatment EEG features to predict TMS therapeutic outcomes for insomnia. TMS treatment can change brain waves, and the model is expected to be applied clinically, though with limitations such as small sample size and insufficiently detailed brain region division.

## 1 Introduction

Insomnia (ID) is the most common clinical disorder afflicting people of all ages, races, and social classes. Epidemiologic surveys show that more than 40% of the population suffers from insomnia, with 47% of them suffering from voluntary sleep deprivation (SD). Insomnia affects the entire body, from immunity to the mind. Sleep and immunity are linked in both directions. Sleep reduces the risk of infection and improves infection outcomes and vaccination responses. Sleep deprivation leads to chronic, systemic low-grade inflammation and is associated with a variety of diseases with an inflammatory component, including diabetes, atherosclerosis, and neurodegeneration (Besedovsky et al., 2019). Short sleep is associated with atherosclerosis, which is directly linked to the risk of cardiovascular disease (Huang et al., 2023; Baranwal et al., 2023), and a higher risk of diabetes (Huang et al., 2023; Jin et al., 2023). Sleep is directly related to memory and mood regulation, and insomnia not only leads to mood disorders such as anxiety, depression, and possibly even mania but also increases the risk of Alzheimer’s disease.

Transcranial Magnetic Stimulation (TMS) is a non-invasive physical therapy, the main principle is that the pulsed magnetic field generated when an electric current is passed through a coil is applied non-invasively through the skull to the corresponding cortical area. This will change the membrane potential of the cells in the cerebral cortex, generating an induced current that ultimately affects the metabolism and electrical activity of neurotransmitters in the brain, thereby treating neurological disorders by inducing the reconstruction of neural networks and regulating the secretion of various neurotransmitters. Low-frequency transcranial magnetic stimulation can relieve and treat insomnia symptoms (Feng et al., 2019). An analysis of transcranial magnetic stimulation and insomnia showed that transcranial magnetic therapy is effective and safe for treating CID (Jiang et al., 2019; Huang et al., 2018). Meta-studies have also shown that transcranial magnetic stimulation therapy is effective in improving insomnia in patients with CID (Sun et al., 2021; Ma et al., 2021), whether the treatment time is short or long, and can effectively reduce Pittsburgh sleep quality index (PSQI) scores and improve insomnia in patients (Huang et al., 2018; Wu et al., 2021).

To study changes in the nervous system of the brain, EEG signals provide valuable information through the oscillatory dynamics of brain waves (Barkley and Baumgartner, 2003). EEG generally refers to scalp EEG, which is used to record the electric potential of the entire scalp generated by electrical activity in the brain, and there are advantages of high temporal resolution non-invasiveness. In the last 10 years, studies about sleep disorders’ EEG paid more attention to classification, both diseases and sleep stages. Sharma used a support vector machine getting a 78.0% average accuracy in all sleep disorder classification (Sharma et al., 2021), and accuracy of 83.0, 78.0, 77.0, and 72.0% in healthy people, narcolepsy, restless legs syndrome, insomnia, and rapid eye movement disorder (Sharma et al., 2021). In sleep stage classification, the team of Kinoshita received the result of 76.9% sensitivity, and 61.2% accuracy using the RUS-Boost classifier (Kinoshita et al., 2020). Some EEG analysis methods, such as power spectral density (PSD) used in sleep disorder studies. A meta-analysis of EEG spectral analysis suggested that patients with ID have a significant increase in EEG, especially beta and theta band power in resting-state wakefulness (Zhao et al., 2021).

To study the changes in the brain’s nervous system, EEG signals can provide valuable information through the oscillatory dynamics of brain waves (Barkley and Baumgartner, 2003). EEG, generally referred to as scalp electroencephalography, is used to record electrical potentials across the scalp generated by the electrical activity of the brain, and has the advantages of high temporal resolution and non-invasiveness. In the last decade, EEG studies on sleep disorders have focused more on classifying disorders and sleep stages. Sharma classified all sleep disorders using support vector machines with an average accuracy of 78.0% (Sharma et al., 2021) and 83.0, 78.0, 77.0, and 72.0% for healthy individuals, narcolepsy, restless legs syndrome, insomnia, and REM sleep disorder (Sharma et al., 2021), respectively. For sleep stage classification, Kinoshita’s team obtained 76.9% sensitivity and 61.2% accuracy using the RUS-Boost classifier (Kinoshita et al., 2020). Some EEG analysis methods such as power spectral density (PSD) have been used in sleep disorder research. A meta-analysis of EEG spectral analysis showed a significant increase in beta and theta band power in the EEG of patients with ID, especially in the resting wakefulness state (Zhao et al., 2021).

## 2 Method

In this study, we analyzed the effector brain areas and therapeutic effects of TMS treatment by daytime awake and closed-eye EEG of insomnia patients before and after TMS treatment and proposed a prediction model for the prognosis of TMS treatment.

TMS treatment was referred to Stanford Accelerated Intelligent Neuromodulation Therapy (SAINT). TMS stimulation site right dorsolateral prefrontal cortex, stimulation frequency intensity: 1 Hz/80%MT, number of pulses/number of pulses: 1500/10, treatment duration: 7 days. The main technical processes are as follows:

### 2.1 EEG data

We collected pre- and post-treatment EEG data from a total of 15 insomnia patients as resting EEG in awake, quiet, and closed-eye states for 5 min. All patients were from the Neurology Clinic of the Second Affiliated Hospital of Air Force Military Medical University. Inclusion criteria: (1) Age greater than 18 years old; (2) patients with chronic insomnia disorder meeting the diagnostic criteria of ICSD-3; (3) able to complete the assessment of various scales according to clinical needs; (4) able to complete the treatment; (5) informed consent to participate in the clinical EEG study. Exclusion criteria: (1) patients who cannot complete the self-assessment questionnaire; (2) patients who refuse to participate in this study; (3) patients who the researcher considers to be inappropriate to participate in this study; (4) organic brain diseases such as traumatic brain injury, intracranial tumors, infections, etc.; (5) patients with psychiatric disorders such as anxiety and depression, bipolar disorder, etc.; and (6) patients with stents in their body and other patients unable to undergo transcranial magnetic therapy.

The scale used was the Insomnia Severity Index (ISI) scale with the nuclear-modified PSQI scale. The modification was to change the period from 1 month to 1 week, to assess the sleep changes of the patients in 1 week of treatment. The sleep quality assessment after 1 month was also performed using the original PSQI scale.

This study was ethically reviewed by the Second Affiliated Hospital of Air Force Military Medical University (ID: 202308-19). All the EEG data were collected and completed using a Delikai EEG instrument.

Then, we completed the pre-treatment of EEG, including filtering and ICA, with the EEGLAB toolbox (Delorme and Makeig, 2004) (2021.1, Arnaud Delorme and Scott Makeig, CA, United States) and Matlab software (2017b, MathWorks Company, Natick, MA, United States). The filtering parameters are 0–45 Hz, segmented according to a 1 s time window, and the artifacts are removed by ICA, after which the process proceeds to the next step.

### 2.2 Feature extraction

This study used approximate entropy, sample entropy, alignment entropy, power spectral density, median, mean, kurtosis, and skewness.

#### 2.2.1 Entropy analysis

Entropy is a means of information analysis that indicates the degree of chaos or disorder in a system. It was first proposed in Clause Shannon in his paper “Mathematical Principles of Communication” in 1948 (Shannon, 1984). Three entropy value analyses were used in this study, namely approximate entropy, sample entropy, and arrangement entropy.

#### 2.2.2 Power spectral density

PSD, known as the power spectrum, represents the signal power within a unit frequency band. The PSD shows the changes in signal power by frequency, that is, the power distribution of the signal in the frequency domain. The basic definition of PSD can be expressed as:


(1) P=1T∫T/2−T/2f2(t)dt

In Equation 1, P represents the average power of power signal f (t) over the period [−T/2, T/2]. Additionally, the unit of PSD is V2/Hz. To reduce the bias during PSD analysis, Pwelch’s method (Welch, 1967) was used in the experiment.

#### 2.2.3 Linear feature extraction

Some linear features were used in signal processing in the time-frequency domain, such as median, mean, skewness, and kurtosis.

#### 2.2.4 Frequency bands

We divided all EEG data into delta band (1 ~ 4 Hz); theta band (4 ~ 8 Hz); alpha band (8 ~ 13 Hz); beta band (13 ~ 30 Hz); and gamma band (30 ~ 45 Hz) according to the common frequency bands. And calculate the above 8 eigenvalues under 5 frequency bands.

### 2.3 Significant value analysis

We performed paired-sample t-tests for each indicator separately to examine whether the difference between the above indicators before and after treatment was significant and plotted the average inter-subject topography of the above 40 indicators.

We calculated the correlation coefficients between the pre-and post-differences of the above 40 indicators and the pre-and post-differences of the ISI scale and the PSQI scale. The correlation coefficients were obtained to analyze the *r*-value and *p*-value data, and the topographic maps were plotted.

### 2.4 Model construction

We obtain the indicators that are significantly correlated with the ISI scale and PSQI scale pre- and post-score differences by calculating the correlation coefficients between the difference before and after treatment of each indicator and the ISI scale pre- and post-score differences, and the PSQI scale pre- and post-score differences, respectively.

For the ISI scale, the pre-and post-score differences of the significant indicators were used to predict the pre- and post-score differences of the ISI scale scores, modeled as a support vector regression model. Further, we used a more rigorous approach to test whether the SVR model could be used to predict ISI scale pre-post differences. This was done by randomly disrupting the predictor variables between subjects and later re-examining the correlation coefficients between the predicted ISI scale pre-post differences and the true ISI scale pre-post differences using the SVR model and the leave-one-out method on the randomly disrupted data. This process was repeated for 5,000 to obtain 5,000 correlation coefficients. These 5,000 correlation coefficients can form distribution, and this part can be considered as the distribution of correlation coefficients if the null hypothesis (i.e., the predicted values of SVR model are not significantly correlated with the true values) is satisfied, so the percentile of the correlation coefficients of the predicted pre- and post-differences of the ISI scale with the true pre- and post-differences of the ISI scale for the original undisturbed scenario in the above distribution can be taken as a *p*-value for this correlation coefficient. The constructed model was considered predictive if *p* < 0.05.

The same method was used for modeling the PSQI scale.

## 3 Results

### 3.1 Base information

A total of 15 subjects, including 8 females and 7 males, with a mean age of 42 years, were enrolled in this study. All subjects received 7 consecutive days of transcranial magnetic stimulation, targeting the right prefrontal lobe, stimulation frequency intensity: 1 Hz/80%MT, number of pulses/number of times: 1,500/10. The results are shown in the Table 1 below. Subject 10’s PSQI worsened (9 → 15). After TMS treatment, we asked about this result, he did not tell the reason; after 1 month, he told us because of quarrel between couple, actually he had great sleep in recently 1 month. So we kept his EEG data.

**Table 1:** All participants’ basic information.

Subject	Age	PSQI-before	ISI-before	PSQI-after	ISI-after
1	56	14	15	10	11
2	39	16	28	12	18
3	18	12	20	10	15
4	47	14	12	8	10
5	38	17	20	15	16
6	53	18	23	16	15
7	33	15	26	10	16
8	44	18	18	10	13
9	38	11	8	9	7
10	56	9	9	15	15
11	42	18	27	14	16
12	47	17	17	11	8
13	37	14	13	12	11
14	32	18	21	16	17
15	50	19	28	13	14
Average	42	15.3333	19	12.0667	13.4667

### 3.2 EEG feature results

We used five eigenvalues to describe the EEG data and analyzed the eigenvalues for each channel in each of the five EEG frequency bands. We compared changes before and after treatment and determined whether these changes were statistically significant by t-test. As shown in the Table 2 below, we selected statistically significant indicator eigenvalues (*p* < 0.05) for all outcomes for further analysis.

**Table 2:** The result of *t*-test between PSQI + ISI and features.

Feature	PSQI + ISI			
	Frequency bands	Channel	*t*	*p*
ApEn	Gamma	P4	−2.449	0.018
	Delta	Fp2	2.171	0.229
		F4	1.921	0.044
		C3	2.074	0.034
		P3	2.009	0.038
		O1	1.846	0.049
		T5	1.971	0.04
	Theta	Fp2	2.365	0.021
		P3	2.044	0.036
		T3	2.119	0.032
	Alpha	T3	2.038	0.036
		T6	1.833	0.049
sampEn	Delta	F4	1.851	0.049
		F8	1.973	0.04
	Beta	C4	−2.101	0.033
		O1	−2.725	0.012
		F8	−2.021	0.037
	Gamma	F3	−1.892	0.046
PE	Delta	Fp2	2.047	0.036
		P3	2.438	0.019
		P4	2.179	0.029
		O1	2.996	0.008
		F7	2.081	0.034
		T3	1.989	0.039
		T5	2.475	0.018
	Alpha	P3	−1.944	0.042
	Beta	F4	−2.602	0.014
	Gamma	F3	−2.173	0.029
PSD	Alpha	Fp1	1.96	0.041
		Fp2	2.56	0.015
	Beta	F3	2.342	0.022
		T3	2.414	0.02
		T5	2.415	0.02
Skewness	Gamma	T6	−2.921	0.009
Kurtosis	Delta	F4	−2.307	0.023
	Theta	P3	−2.021	0.037
	Gamma	T6	−2.968	0.008

### 3.3 Relationship with treatment scores

**Figure 1:**
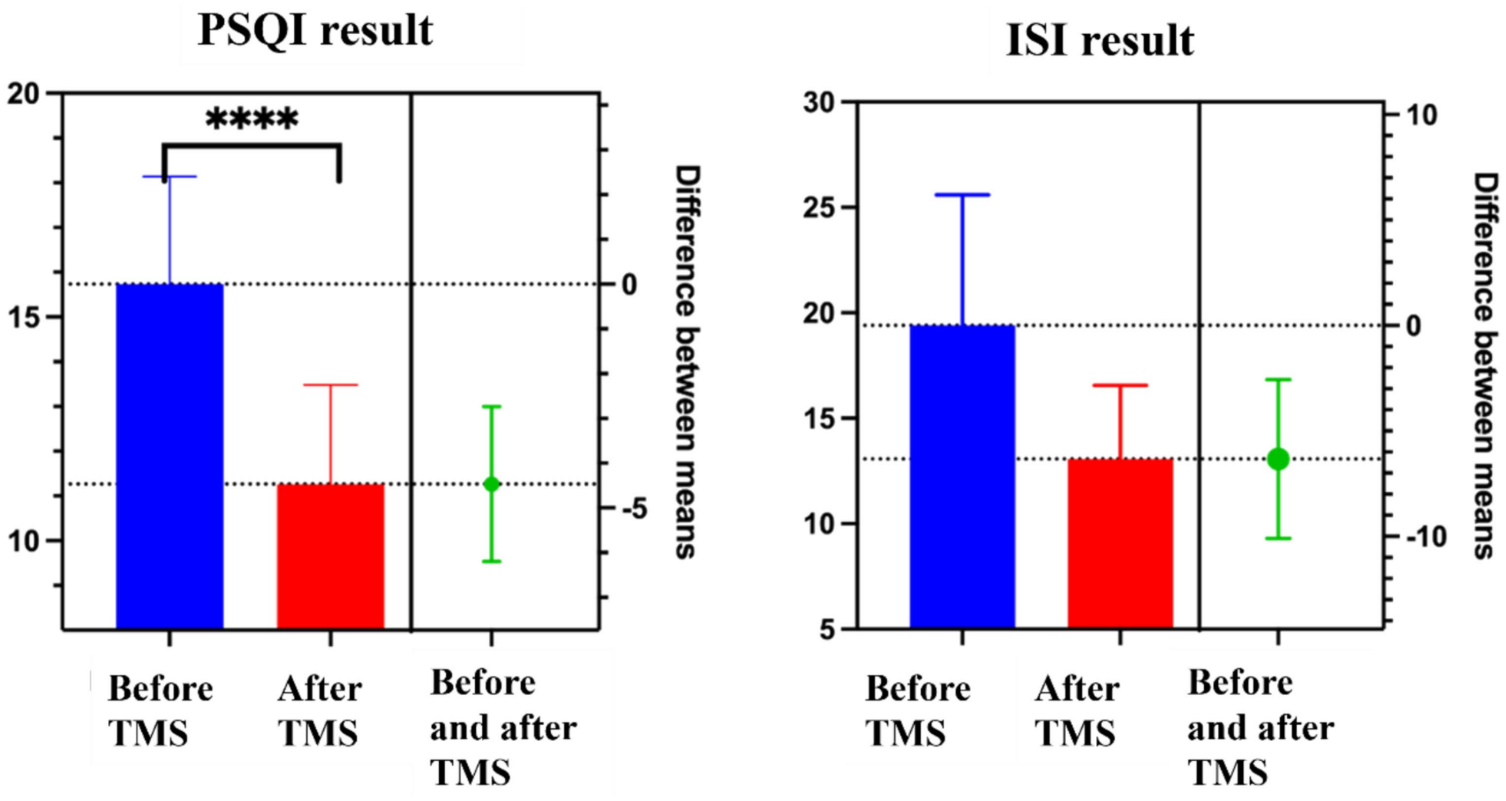
The result of *t*-test between before and after treatment in PSQI/ISI.

In this study, we compared the PSQI total score and ISI total score before and after transcranial magnetic stimulation treatment, and the difference between the before and after comparisons was statistically significant (*p* < 0.05) as calculated by paired *t*-test (Table 3; Figure 1), which indicates that transcranial magnetic stimulation treatment has a certain therapeutic effect on chronic insomnia.

**Table 3:** The result of *t*-test between before and after treatment in PSQI/ISI.

	PSQI	ISI
Before	15.333	19
After	12.067	13.467
*p*	<0.005	<0.005

Next, we calculated the correlation coefficients between the before-and-after differences of the above 40 indicators the before-and-after differences of the ISI scale, and the before-and-after differences of the PSQI scale. The following topographic map was drawn. We selected the characteristic values of the indicators that were statistically significant (*p* < 0.05) in all the results in the following Tables 4, 5 for further analysis.

**Table 4:** The result of correlation coefficients between PSQI and features.

Feature	PSQI			
	Frequency bands	Channel	*r*_value	*p*_value
ApEn	Theta	O2	0.689	0.028
	Alpha	P3	0.662	0.037
		T3	0.745	0.014
sampEn	Gamma	P3	0.922	<0.001
PE	Beta	T4	−0.745	0.013
Skewness	Alpha	P3	−0.808	0.005
	Gamma	P3	−0.761	0.011
		O1	−0.664	0.036
		F8	−0.649	0.042
Kurtosis	Theta	P3	0.735	0.016
	Alpha	P3	−0.814	0.004
	Gamma	P3	−0.738	0.015
		O1	−0.679	0.031
		F8	−0.635	0.049

**Table 5:** The result of correlation coefficients between ISI and features.

Feature	ISI			
	Frequency bands	Channel	*r*_value	*p*_value
ApEn	Alpha	P4	0.718	0.02
		O2	0.689	0.027
		T3	0.75	0.013
sampEn	Gamma	P3	0.658	0.039
PE	Beta	T4	−0.926	<0.001
PSD	Beta	T3	0.639	0.047
Skewness	Delta	F7	−0.685	0.029
	Beta	O1	−0.642	0.046
	Gamma	O1	−0.835	0.003
Kurtosis	Beta	O1	−0.643	0.045
	Gamma	O1	−0.838	0.003

### 3.4 Model construction

An SVR model was constructed as described in the research methodology. In predicting the pre-post differences of the ISI scale using the SVR model and the leave-blank method, we found that the correlation coefficient between the predicted pre-post differences of the ISI scale and the true pre-post differences of the ISI scale was 0.7805, with a *p*-value of 0.0077. After further training, we obtained a *p*-value of 0.0150 < 0.05, indicating that the constructed SVR model can be used to predict the pre- and post-differences of the ISI scale.

The same is true for the PSQI prediction model. The correlation coefficient between the pre-and post-differences of the predicted PSQI scales and the pre-and post-differences of the true PSQI scales was 0.7136, with a *p*-value of 0.0205. After further rigorous training, we obtained a *p*-value of 0.0396 < 0.05, which suggests that the constructed SVR model can be used for predicting pre-post differences in PSQI scales.

## 4 Discussion

In this study, we used awake and closed-eye EEGs of insomnia patients before and after TMS treatment to discover the brain regions that changed before and after treatment by methods of eigenvalue extraction, significance analysis, and model construction, and successfully constructed a prognostic prediction model for insomnia TMS treatment. We built an SVR prediction model by using awake and closed-eye EEGs of 15 insomnia patients before and after TMS treatment for 7 days, through the significance differences of 8 eigenvalues in 5 EEG frequency bands, i.e., proximity entropy, sample entropy, alignment entropy, power spectral density, median, mean, kurtosis, and skewness. The model combines the PSQI and ISI scale scores before and after treatment and can be used to predict the prognosis of TMS treatment using the eigenvalue differences in the leads.

### 4.1 Insomnia treatment discussion

Insomnia brain area research has been a hot spot in insomnia research. Previous studies have mainly focused on the prefrontal cortex (Lee et al., 2022; Yuan et al., 2020), and related studies in recent years have gradually focused on the alteration and increase/decrease of the functional connectivity between the prefrontal cortex and other brain regions (Fasiello et al., 2022), such as the amygdala (Kweon et al., 2023; Liu et al., 2024) and cerebellum (Lin et al., 2023). In this study, we distinguished the brain regions of insomnia patients by the regions corresponding to the EEG leads. Among the different brain regions, we found that in the fast-wave frequency band, the changes in F7 were the most obvious; the F7 lead was located in the prefrontal area, which was also the direct area of TMS stimulation in this study. This implies that transcranial magnetic stimulation treatment did reduce the appearance of fast waves in the prefrontal region of insomnia patients (Zhou et al., 2023); relevant literature suggests that an increase in fast waves inhibits the production and maintenance of sleep, which may be related to the improvement of insomnia. On the contrary, slow wave is an important component of sleep and is the main component of NREM3 sleep, and its appearance means that the patients entered deep sleep (McConnell et al., 2022), before and after the treatment, the frequency of slow waves increased, which suggests that the deep sleep of the insomnia patients has improved. The increase in the slow wave frequency in the temporal lobe region was the most significant after the treatment, which means that the sleep quality of insomnia patients was improved after TMS treatment, and the improvement of long-term memory function may be related to this (Han et al., 2023).

### 4.2 SVR and other artificial intelligence models

The prognostic predictive modeling of TMS for insomnia was implemented by the SVR model. In this study, outcomes were predicted by scores on two insomnia scales. For the results of the Modified PSQI scale and the ISI scale, a reduction of 25% or more was considered valid, and after 1 week of TMS treatment, the treatment efficacy rate was approximately 50% for the Modified PSQI scale and 80% for the ISI scale, with a combined efficacy rate of 100% for both scales. Combining the scale scores of the 15 subjects, there was a statistically significant decrease in both the modified PSQI and ISI after 1 week of TMS treatment. This demonstrated that 1 week of transcranial magnetic stimulation treatment could improve sleep duration and sleep quality in insomnia patients. Subsequently, we combined the Brain Area Scale and Sleep Scale with the SVR model and performed hundreds of data entry simulations, obtaining a *p*-value of less than 0.05, which demonstrated that the constructed SVR model can be used to predict the scale differences before and after TMS treatment. In the future, we hope that more data and more detailed classification of brain regions will be applied to the training of this model to provide the possibility of prediction for clinical work.

Among the sleep-related artificial intelligence models, the most common is automatic sleep staging, which performs sleep staging individually or jointly through sleep EEG, EMG, ECG respiratory rhythm data, etc. Currently, the research of related algorithms for classification mainly adopts methods such as machine learning and artificial intelligence. The current common sleep staging algorithms are (1) Gradient-weighted Class Activation Mapping (Grad-CAM), an explainable artificial intelligence (XAI) algorithm, obtained a correlation coefficient of 0.772, and an accuracy of 86.9% (Vaquerizo-Villar et al., 2023). (2) Several research teams have analyzed PSG data by Consumer Sleep Technologies (CST) methodology, with the highest accuracy of 89.65%, and five studies exceeded 90% accuracy in wake/sleep classification, with the highest being 96%; for the classification of five stages of sleep (wakefulness, N1, N2, N3, and R) without sacrificing the other sleep stages, the highest N1% was achieved using the CST method based on the CNN framework, with an accuracy of 80.2% (Shao et al., 2024). (3) When the multiple intelligent models of random forests, decision trees, and linear discriminant analysis were used for staging, the sensitivity, specificity, and accuracy detection performance were 96.0, 94.0, and 96.0% (Tripathi et al., 2022), respectively. Many of the methods and models mentioned above are gradually being applied in finished software to help clinicians with sleep staging. There are also AI models dedicated to sleep disorder disease differentiation. Recognizing and classifying respiratory events, constructing hypnograms of patients thus differentiating OSA patients with an accuracy of 92.31% (Cabrero-Canosa et al., 2003). A Meta-analysis article on intelligent algorithms for insomnia summarizes nearly 10 years of literature and summarizes about 15 algorithms, from the CNN model with the highest accuracy rate of 98.91% to the Support Vector Machine model with an accuracy rate of 76%, and the Random Forest model with an accuracy rate of 74% (Ingle et al., 2024), which is a multi-dimensional summary of common models for insomnia classification. Regardless of the model, it has advanced the progress of insomnia classification models. Meanwhile, intelligent algorithms are applied to daily life. A company statistically classified night sleep, sleep habits, and daily habits of insomnia patients through CAE and cluster analysis of continuous data, and identified five new clusters of participants in insomnia activities that could not be identified by traditional methods, and there were significant differences in sleep and behavioral characteristics between the groups, which put forward a new idea for the individualized treatment of insomnia (Park et al., 2019). Regardless of the model, which focuses on diagnosis, there is a lack of predictive models for the prognosis of insomnia treatment. In this study, based on the sleep scale score as a treatment outcome, an SVR treatment prediction model with a *p*-value < 0.05 was obtained by constructing the SVR model and hundreds of data input simulations. The results of the study illustrate that the constructed SVR model can be used to predict the scale difference before and after TMS treatment, i.e., it can predict the treatment prognosis of insomnia. In the future, we hope that more data and more detailed brain area classification can be applied to the training of this model, which can provide a prediction of treatment outcomes for clinical work.

### 4.3 Shortcomings and future work

In this study, we proposed a prognostic prediction model for TMS treatment, and also briefly studied the brain regions that respond to TMS treatment. However, there are still some shortcomings: (1) Only 15 subjects were included in this study. (2) The 16-lead EEG used in this study could not break down the brain regions in detail. We hope that in the future we can: (1) Expand sample size (*n* ≥ 50) and include a control group. (2) Upgrade EEG resolution (64+ channels) to better localize neural activity. (3) Validate the model prospectively in real-world settings. (4) Explore advanced algorithms (e.g., deep learning) to improve predictive accuracy.

## 5 Conclusion

In conclusion, this study found that after 1 week of transcranial magnetic stimulation treatment, insomnia patients showed a decrease in fast waves in the prefrontal region and an increase in slow waves in the hippocampus-amygdala region, which may be related to the improvement of insomnia. The study also constructed a prognostic prediction model for TMS treatment of insomnia, which is expected to be applied in clinical work.

## Data Availability

The raw data supporting the conclusions of this article will be made available by the authors, without undue reservation.
